# Survival and Short-Term Morbidities Among Extremely Preterm Infants: A Prospective Cohort Study

**DOI:** 10.7759/cureus.91703

**Published:** 2025-09-06

**Authors:** Shikha Khandelwal, Vikram Datta

**Affiliations:** 1 Neonatology, Pandit Bhagwat Dayal Sharma Post Graduate Institute of Medical Sciences, Rohtak, IND; 2 Neonatology, Lady Hardinge Medical College, Delhi, IND

**Keywords:** bronchopulmonary dysplasia, extremely preterm infants, india, intraventricular hemorrhage, necrotizing enterocolitis, neonatal outcomes, survival

## Abstract

Background: Outcomes of extremely preterm (EPT) infants in low- and middle-income countries (LMIC) remain heterogeneous, with limited gestational age (GA)-defined data from India.

Objective: To evaluate survival, short-term morbidities, and predictors of mortality among infants born ≤28 weeks’ gestation in a North Indian tertiary neonatal intensive care unit (NICU).

Methods: This prospective observational cohort included all inborn infants ≤28 weeks admitted between November 2020 and October 2021, excluding those with major anomalies. Maternal, perinatal, and neonatal data were collected prospectively. The primary outcome was survival to discharge; secondary outcomes included major morbidities. Predictors of mortality were assessed using multivariable logistic regression.

Results: Eighty-three infants were enrolled (median GA 27.0 weeks (IQR 26.3-27.9); median birth weight 892 g (IQR 731-1021)). Overall survival to discharge was 61.4%, with survival increasing with gestational age. Sepsis (37.5%) and extreme prematurity (40%) were leading causes of death. Major morbidities included sepsis (61.4%), bronchopulmonary dysplasia (BPD) at 36 weeks postmenstrual age (PMA) (41.5% any grade, 15.1% moderate-severe), necrotizing enterocolitis (NEC) ≥ stage II (13.3%), severe intraventricular hemorrhage (IVH) (12.0%), and treatment-requiring retinopathy of prematurity (ROP) (7.8%). In adjusted analysis, higher birth weight (adjusted odds ratio (AOR) 0.59 per 100 g increase; 95% CI 0.37-0.94) and higher five-minute Apgar score (aOR 0.25 per point; 95% CI 0.13-0.48) independently predicted survival. The final model demonstrated excellent discrimination (area under the curve (AUC) 0.89, 95% CI 0.83-0.96).

Conclusions: Survival of EPT infants in this Indian cohort was higher than pooled LMIC estimates but remained lower than high-income benchmarks. Birth weight and five-minute Apgar score were the strongest predictors of outcome. Strengthening perinatal care, infection prevention, and standardized neonatal practices may further improve outcomes.

## Introduction

Prematurity is the leading cause of neonatal mortality. Outcomes of extremely preterm neonates have improved substantially in high-income countries over recent decades, with survival rates exceeding 80-90% at 27-28 weeks’ gestation [1,2]. However, outcomes remain considerably poorer in low- and middle-income countries, where resource constraints and variability in perinatal care contribute to higher mortality [3]. In India, most published data describe extremely low birth weight (ELBW) cohorts rather than infants <28 weeks’ gestation, and outcomes remain heterogeneous [3-6]. Understanding these outcomes is essential to benchmark care, identify modifiable risk factors, and guide resource allocation in neonatal intensive care units (NICUs). We aimed to evaluate survival, morbidities, and predictors of mortality in a prospective cohort of ≤28-week infants in a North Indian tertiary NICU.

## Materials and methods

Study design and setting

This prospective observational study was conducted in the Level 3 NICU of a tertiary care government centre in Delhi, India, from November 2020 to October 2021.

Participants

All inborn neonates born at ≤28 weeks of gestation and admitted to the NICU during the study period were eligible. Infants with major congenital anomalies were excluded. As per unit policy, active treatment was given to all newborns born ≥25 weeks and >500 grams. Active treatment between 23 and 25 weeks’ gestation was undertaken after detailed counselling and parental consent, and included full resuscitation at birth with invasive ventilation, surfactant administration, provision of total parenteral nutrition, and other intensive interventions such as blood product transfusion and management of shock as required. No active treatment was offered below this gestation. Written informed consent was obtained from parents, and the study was approved by the Institutional Ethics Committee (approval LHMC/IEC/2019/40).

Data collection and definitions

Maternal and perinatal details, including antenatal corticosteroid (ACS) exposure, mode of delivery, and maternal complications such as preeclampsia, clinical chorioamnionitis, and premature rupture of membranes, were recorded. Neonatal variables included gestational age (GA), birth weight (BW), sex, Apgar scores, and resuscitation at birth. The clinical course, respiratory support, major morbidities, and outcomes were documented prospectively.

GA was determined from first-trimester ultrasound or last menstrual period. Small for gestational age (SGA) was defined as BW below the 10th centile on Fenton charts [7]. Hemodynamically significant patent ductus arteriosus (hsPDA) was diagnosed based on echocardiographic evidence in conjunction with clinical signs. Bronchopulmonary dysplasia (BPD) was defined according to the National Institutes of Health (NIH) consensus criteria and graded at 36 weeks’ postmenstrual age (PMA) [8]. Necrotizing enterocolitis (NEC) was staged using Bell’s criteria [9], while intraventricular hemorrhage (IVH) was classified using the Papile grading system [10]. Two composite outcomes were analyzed: (i) any BPD or death before 36 weeks PMA, and (ii) moderate-to-severe BPD or death.

Outcomes

The primary outcome was survival to hospital discharge. Secondary outcomes included major neonatal morbidities (BPD, NEC, severe IVH, retinopathy of prematurity (ROP), sepsis), duration of respiratory support, and length of NICU stay.

Statistical analysis

Continuous variables were expressed as median (interquartile range (IQR)) and compared using the Mann-Whitney U test. Categorical variables were presented as n (%) and compared using Chi-square or Fisher’s exact test, as appropriate. Multivariable logistic regression was performed to identify predictors of mortality, and results were expressed as adjusted odds ratios (aOR) with 95% confidence intervals (CI). Because GA and BW were highly collinear, only BW was included in the multivariable model to avoid instability of estimates. Model fit was assessed using omnibus χ², Nagelkerke R², and classification accuracy. A p-value <0.05 was considered statistically significant. Analyses were performed using SPSS version 20 (IBM Corp., Armonk, NY, USA).

## Results

**Figure 1:**
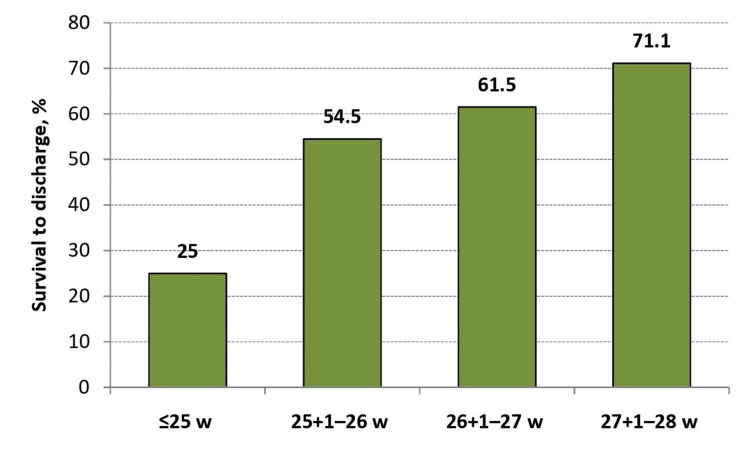
Survival to discharge according to gestational age groups Bar chart showing survival rates to discharge among extremely preterm infants, stratified by gestational age: ≤25 weeks (25.0%, n = 8), 25+1–26 weeks (54.5%, n = 11), 26+1–27 weeks (61.5%, n = 26), and 27+1–28 weeks (71.1%, n = 38). Survival improved progressively with increasing gestational age. Statistical comparison was performed using Fisher’s exact test, with p < 0.05 considered statistically significant.

**Figure 2:**
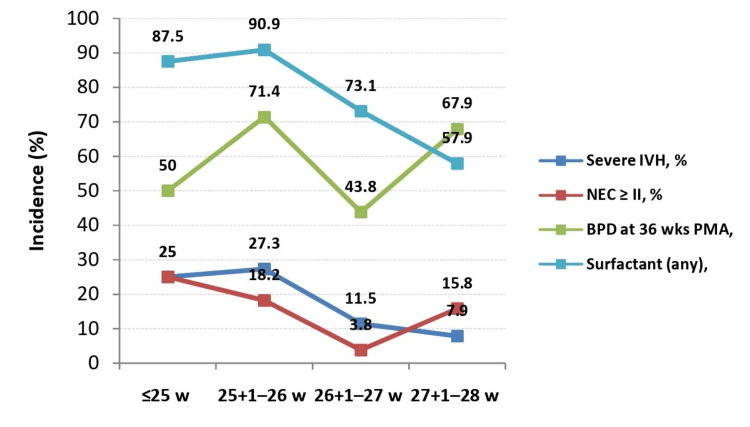
Incidence of major morbidities across gestational age groups Line chart showing the incidence of severe IVH, NEC ≥ stage II, BPD at 36 weeks PMA, and RDS requiring surfactant among extremely preterm infants stratified by gestational age: ≤25 weeks (n = 8), 25+1–26 weeks (n = 11), 26+1–27 weeks (n = 26), and 27+1–28 weeks (n = 38). Statistical comparisons across groups were performed using the Chi-square test or Fisher’s exact test as appropriate, with p < 0.05 considered statistically significant. Abbreviations: RDS, respiratory distress syndrome; IVH, intraventricular hemorrhage; NEC, necrotizing enterocolitis; BPD, bronchopulmonary dysplasia; PMA, postmenstrual age.

A total of 83 extremely preterm infants were enrolled, with a median gestational age of 27.0 weeks (IQR 26.3-27.9; range 24.1-28.0) and median birth weight of 892 g (IQR 731-1021; range 490-1305). Nearly one-fifth were small for gestational age, and 79.5% received at least one dose of antenatal corticosteroids (Table 1). Overall survival to discharge was 61.4%, with higher survival at increasing gestational ages (Figure 1). The distribution of major morbidities also varied by gestational age: the incidence of BPD at 36 weeks’ PMA, severe IVH (grade 3 or 4), NEC ≥ Stage II, and surfactant use is shown in Figure 2. The primary causes of mortality were extreme prematurity (40%) and sepsis (37.5%). Major morbidities and outcomes are summarized in Table 2, and respiratory support and interventions in Table 3.

**Table 1:** Baseline Characteristics of Extremely Preterm Infants (n = 83) ᵃ Includes 2 triplet and 10 twin pregnancies (71 deliveries in total, 2 intrauterine deaths). ᵇ Denominator = deliveries (n = 71). ᶜ Defined as ≥1 dose of antenatal corticosteroid ᵈ Highest level of resuscitation required Abbreviations: IQR, interquartile range; ACS, antenatal corticosteroids; PPV, positive pressure ventilation.

Variable	n (%) or Median (IQR)
Gestational age, weeks	27 (26.3–27.9)
Birth weight, g	892 (731–1021)
Male sex	45 (54.2)
Small for gestational age (Fenton <10th %)	16 (19.3)
Multiple gestation	24 (28.9)ᵃ
Mode of delivery (Cesarean section)	17 (23.9)ᵇ
Antenatal corticosteroids (any) ᶜ	66 (79.5)
Complete ACS course	34 (40.9)
Maternal preeclampsia	6 (8.5)ᵇ
Maternal chorioamnionitis (clinical)	5 (7.0)ᵇ
Preterm premature rupture of membranes >18 h	30 (36.1)
Apgar at 5 min	7 (6–8)
Resuscitation at birth (PPV/intubation)	36 (43.4)ᵈ

**Table 2:** Major Morbidities and Outcomes in Extremely Preterm Infants (n = 83) ᵃ Includes both sepsis screen positive and/or culture positive. ᵇ Denominator excludes infants who died before first ROP screening (n = 64). ᶜ Denominator restricted to infants who survived to 36 weeks PMA (n = 53). ᵈ Composite outcomes calculated for the entire cohort (n = 83). Abbreviations: IQR, interquartile range; BPD, bronchopulmonary dysplasia; PMA, postmenstrual age; NICU, neonatal intensive care unit; ROP, retinopathy of prematurity.

Variable	n (%) or Median (IQR)
Sepsisᵃ	
• Early-onset sepsis	16 (19.3)
• Late-onset sepsis	35 (42.2)
Necrotizing enterocolitis ≥ Stage II (Bell’s)	11 (13.3)
Intraventricular hemorrhage Grade III–IV (Papile)	10 (12.0)
Retinopathy of prematurity requiring treatmentᵇ	5 (7.8)
BPD at 36 weeks PMAᶜ	
• Any grade	22 (41.5)
• Moderate–severe	8 (15.1)
Composite outcomesᵈ	
• Any BPD or death before 36 weeks PMA	52 (62.6)
• Moderate–severe BPD or death	38 (45.8)
Survival to discharge	51 (61.4)
Mortality (all-cause)	32 (38.6)
Length of NICU stay, days	44 (34–63)

**Table 3:** Respiratory Support and Interventions during NICU stay (n = 83) ᵃ First-line respiratory support refers to the initial mode instituted after birth; categories are mutually exclusive. ᵇ Includes both first-line and rescue IMV. ᶜ Calculated among ventilated infants (n = 44). Abbreviations: IQR, interquartile range; CPAP, continuous positive airway pressure; IMV, invasive mechanical ventilation; PDA, patent ductus arteriosus; hsPDA, hemodynamically significant patent ductus arteriosus; NICU, neonatal intensive care unit.

Variable	n (%) or Median (IQR)
Surfactant given (any dose)	58 (69.8)
First-line respiratory supportᵃ	
• CPAP	24 (28.9)
• Non-invasive ventilation	32 (38.6)
• Invasive mechanical ventilation (IMV)	27 (32.5)
Invasive ventilation (any, at any time)ᵇ	44 (53.0)
Duration of IMV, days	3 (2–6)ᶜ
Caffeine therapy	77 (92.8)
Hemodynamically significant PDA	31 (37.3)
Treatment for hsPDA (drug/surgery)	24 (28.9)

**Figure 3:**
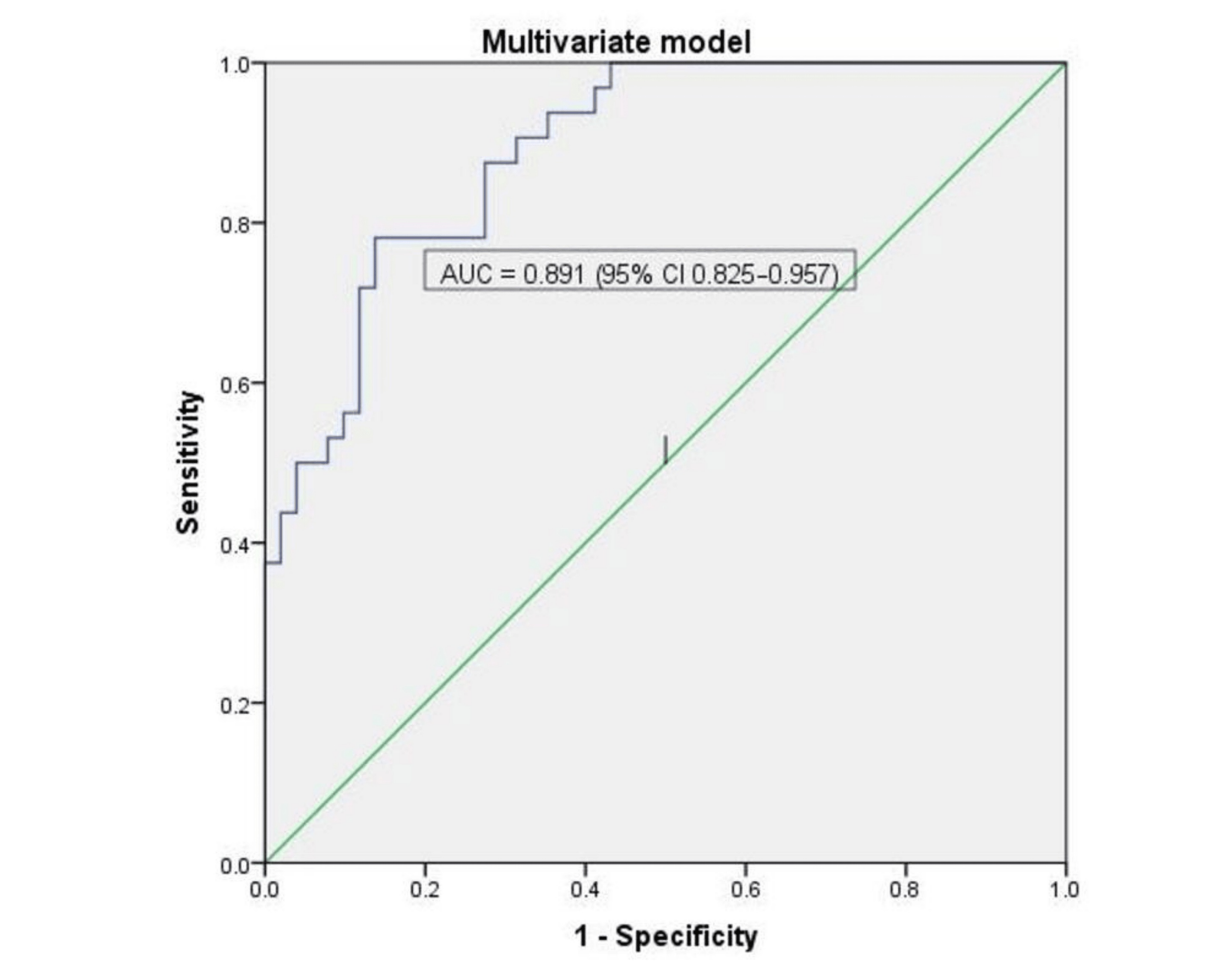
Receiver Operating Characteristic (ROC) Curve of the Multivariate Logistic Regression Model The ROC curve demonstrates the discriminative ability of the multivariate model to predict the outcome. The area under the curve (AUC) was 0.891 (95% CI 0.825–0.957), indicating excellent predictive performance. The diagonal green line represents the reference line (AUC = 0.5), corresponding to no discriminative ability.

In multivariable analysis, only higher birth weight and five-minute Apgar score independently predicted survival, while antenatal steroid coverage, male sex, and SGA status were not significant (Table 4). Each 100-g increase in birth weight reduced the odds of death by 41% (aOR 0.59, 95% CI 0.37-0.94; p = 0.025), and each one-point increase in Apgar score decreased the odds by 75% (aOR 0.25, 95% CI 0.13-0.48; p < 0.001). The model showed good overall fit (Omnibus χ² = 44.7, p < 0.001; Nagelkerke R² = 0.57) with 80.7% classification accuracy (sensitivity 71.9%, specificity 86.3%), and excellent discrimination (area under the curve (AUC) 0.89, 95% CI 0.83-0.96; p < 0.001) (Figure 3).

**Table 4:** Multivariable Logistic Regression for Predictors of Mortality in Extremely Preterm Infants (n = 83) Multivariable logistic regression analysis showing adjusted odds ratios (aOR) with 95% confidence intervals (CI) for predictors of mortality. Birth weight and Apgar score at 5 minutes were independently associated with reduced risk of mortality. Model fit statistics: Omnibus χ²(6) = 44.7, p < 0.001; Nagelkerke R² = 0.57; classification accuracy = 80.7% (sensitivity 71.9%, specificity 86.3%). A p-value <0.05 was considered statistically significant.

Predictor	aOR	95% CI	p-value
Birth weight (per 100 g ↑)	0.59	0.37 – 0.94	0.025
Apgar score at 5 min (per 1-point ↑)	0.25	0.13 – 0.48	<0.001
Antenatal steroids – Partial vs Full	0.95	0.16 – 5.6	0.95
Antenatal steroids – None vs Full	0.61	0.10 – 3.8	0.58
Male sex	1.53	0.41 – 5.7	0.53
Small for gestational age	1.62	0.32 – 8.2	0.57

## Discussion

In this prospective cohort of 83 inborn infants ≤28 weeks’ gestation, survival to discharge was 61.4%. The median GA (27.0 weeks) and BW (892 g) highlight the inclusion of high-risk, extremely preterm infants representative of Indian tertiary NICUs. Survival in our cohort was higher than pooled estimates from low- and middle-income countries. A meta-analysis of 192 studies (22,278 ELBW and 18,338 extremely low gestational age neonates) reported only 39% pooled survival over two decades in developing nations, with Indian studies contributing similar rates (39%) [3]. The improved outcome in our study may reflect contemporary, single-center tertiary care, compared to older and heterogeneous series. A North Indian ELBW cohort (2013-2014, mean GA 27.9 weeks) reported 62% survival [4], closely mirroring our findings. Earlier Indian reports documented even poorer survival [6,11], consistent with evolving perinatal and neonatal practices. In contrast, survival in high-income settings remains substantially higher: the National Institute of Child Health and Human Development (NICHD) Neonatal Research Network reported 78.3% survival among 22-28 week infants (2013-2018), rising to 94% at 28 weeks [12], while a Singapore network documented 80% survival (19% at 23 weeks vs 93% at 28 weeks [13]. The highest survival and lowest morbidities in extreme preterm neonates have been reported from Japan [14]. These comparisons underscore the gap in outcomes between resource-rich and resource-limited contexts, attributable to differences in antenatal care coverage (including timely administration of steroids), doctor- and nurse-to-patient ratios, availability of advanced neonatal intensive care facilities, higher burden of sepsis, and socioeconomic factors such as parental literacy.

Predictors of mortality in our cohort (lower BW and lower five-minute Apgar) align with both Indian and international data, where GA/BW and early physiologic status dominate risk models and explain much of the survival gradient across gestations and systems [15,16].

Morbidity in our cohort remained substantial. Pulmonary complications, particularly BPD, which affected a sizeable proportion of survivors. The incidences of severe IVH, NEC, and treatment-requiring ROP in our cohort were within ranges reported for similar gestational groups. A pooled developing countries’ meta-analysis described ~14% severe IVH, 8% NEC, 37% BPD, 40% sepsis, and 20% ROP requiring treatment [3] - findings broadly consistent with our observations, though differences in definitions and surveillance methods complicate direct comparison. Even in high-survival networks, pulmonary morbidity persists; for example, the Singapore ≤28-week cohort reported ~67% moderate-severe BPD among survivors [14].

Strengths of our study include its prospective design, use of standardized definitions, and focus on a GA-defined Indian extreme prematurity cohort, which is under-represented compared with BW-based ELBW series. Limitations include the single-center nature, modest sample size (limiting power to detect associations such as antenatal steroid effect), and absence of long-term neurodevelopmental outcomes. Cross-study comparison is further complicated by variability in BPD definitions, thresholds for active intervention at the lowest gestations, and sepsis surveillance practices.

## Conclusions

In conclusion, this prospective study provides contemporary outcomes of a gestational age-defined cohort of extremely preterm infants in India, a group underrepresented in existing literature. Survival in our cohort compares favorably with pooled reports from low- and middle-income countries and Indian studies, but remains below benchmarks from high-income countries. System-level improvements, such as universal antenatal steroid coverage, structured perinatal transfer, standardized early respiratory and nutritional strategies, robust infection-prevention, and development of multicenter benchmarking networks, are priorities. Such measures, proven effective in the Neonatal Research Network of the Eunice Kennedy Shriver NICHD and the International Network for Evaluating Outcomes of Neonates (iNeo) collaborations, hold promise for improving outcomes of India’s most immature infants. Future multicenter studies are needed to validate these findings and guide policy for neonatal intensive care in resource-limited settings.
